# Long-Term Mortality and Medical Burden of Patients with Chronic Obstructive Pulmonary Disease with and without Subsequent Stroke Episodes

**DOI:** 10.3390/ijerph17072550

**Published:** 2020-04-08

**Authors:** Yu-Shu Yen, Dorji Harnod, Cheng-Li Lin, Tomor Harnod, Chia-Hung Kao

**Affiliations:** 1 Department of Neurosurgery, Neurological Institute, Taipei Veterans General Hospital, Taipei 11217, Taiwan; yushuyen@gmail.com; 2 School of Medicine, National Yang-Ming University, Taipei 11221, Taiwan; 3 Department of Emergency and Critical Care Medicine, Fu Jen Catholic University Hospital, Fu Jen Catholic University, New Taipei City 24352, Taiwan; A00635@mail.fjuh.fju.edu.tw; 4 School of Medicine, College of Medicine, Fu Jen Catholic University, New Taipei City 24205, Taiwan; 5 Management Office for Health Data, China Medical University Hospital, Taichung 40447, Taiwan; orangechengli@gmail.com; 6 College of Medicine, China Medical University, Taichung 40402, Taiwan; 7 Department of Neurosurgery, Hualien Tzu Chi Hospital, Buddhist Tzu Chi Medical Foundation, Hualien Hualien 97002, Taiwan; 8 College of Medicine, Tzu Chi University, Hualien 97071, Taiwan; 9 Department of Nuclear Medicine and PET Center, and Center of Augmented Intelligence in Healthcare, China Medical University Hospital, Taichung 40447, Taiwan; 10 Department of Bioinformatics and Medical Engineering, Asia University, Taichung 41354, Taiwan; 11 Graduate Institute of Biomedical Sciences and School of Medicine, College of Medicine, China Medical University, Taichung 40402, Taiwan; 12 Center of Augmented Intelligence in Healthcare, China Medical University Hospital, Taichung 40447, Taiwan

**Keywords:** cohort study, COPD, mortality, National Health Insurance, stroke

## Abstract

Background: We used the Taiwan National Health Insurance Research Database (NHIRD) to determine the differences in mortality and medical burden between patients with chronic obstructive pulmonary disease (COPD) with and without stroke. Methods: We enrolled participants aged ≥20 years and defined four subgroups in this study, namely patients with COPD (International Classification of Diseases, Ninth Revision, Clinical Modification (ICD-9 CM): 491, 492, 494, and 496), patients with COPD with stroke (ICD-9 CM: 430–438), with COPD without stroke, and comparison subgroups. We calculated the hazard ratios and 95% CIs for all-cause mortality risk, average duration of hospitalization, and frequency of medical visits in these subgroups after adjustments were made for age, sex, and comorbidities. All participants were followed until the date of death, the date they were censored, the date they withdrew from the NHIRD, or 31 December, 2013. Results: In total, 9.70% (men vs. women, 11.19% vs. 8.28%) of patients with COPD developed subsequent stroke during the 14 year follow-up. After a stroke, the risk of mortality exhibited a 2.66- to 5.05-fold increase, especially in the younger ones. COPD with stroke was also a leading factor in the increase in the average number of hospitalization days and frequency of medical visits. Conclusion: The mortality risk of patients with COPD is considerably increased by stroke independent of the other effects of COPD. Moreover, the average number of hospitalization days and frequency of medical visits dramatically increased in patients with COPD after stroke.

## 1. Introduction

Chronic obstructive pulmonary disease (COPD) involves damage and inflammation of the lung airways and narrowing of the air sacs. A global increase in the prevalence of COPD has been noted, with approximately 7% to 20% of the adult population reported to currently have this disease across the various countries and populations studied [1,2,3]. According to the 2010 Global Burden of Disease Study, COPD is responsible for approximately 5% of global disability and 5% of total death, making it the fourth most common cause of death in the world [3]. To date, no cure for COPD has been made available, although the current treatment proposed can ameliorate the symptoms and improve the quality of life of patients and prevent acute worsening of the disease. Since the second half of the 20th century, treating COPD has been a challenge for clinicians worldwide, and a burden to the public health care systems [3,4,5]. In 2018, pneumonia and COPD were respectively reported as the third and seventh leading causes of death in Taiwan, an eastern Asian developing country [6]. 

Despite tobacco smoke being a well-known cause of COPD, 20% to 30% of patients who develop COPD have never smoked [7,8]. Secondary and tertiary smoking, occupational exposure to dust, exposure to noxious fumes and vapors, and indoor and outdoor air pollution from the burning of biomass fuels may all predispose an individual to developing COPD, especially among women in developing or underdeveloped countries [9]. Most patients with COPD are middle-aged or elderly adults [1,2,3]. In these age groups, stroke, whether ischemic or hemorrhagic, is another leading life-threatening disease. In most cases, COPD diagnosis is made before patients experience a stroke episode [10]. Nevertheless, COPD itself has been proven to be a predisposing factor for new-onset stroke with as high as a 6.66-fold risk of stroke in patients with COPD [11]. However, we wished to determine how much the mortality and medical burden of patients with COPD increases after stroke. To examine the associations between COPD and stroke, we studied the differences in all-cause mortality risk and medical burden between patients with COPD with and without stroke. 

Because of the similar ethnic and cultural background between Taiwan and numerous developing countries in Asia [12], the findings from our study might be applicable to the development of more efficient medical care systems in other Asian countries. 

## 2. Methods 

### 2.1. Data Source 

The National Health Insurance program in Taiwan was established in 1995, and a nationwide database named the National Health Insurance Research Database (NHIRD) was concurrently built. The database contains data pertaining to individual health claims from the National Health Insurance Program. It is a national database offering comprehensive medical records and a long-term follow-up period. Identification numbers are used to encrypt the information before its release, thus protecting the privacy of each individual included in the study. To conduct this study, we used the Longitudinal Health Insurance Database (LHID), which consists of 1 million patients randomly selected from the NHIRD. Moreover, it is a nationwide database offering comprehensive medical records and a long-term follow-up period. 

The history of diagnoses was coded according to the International Classification of Diseases, Ninth Revision, Clinical Modification (ICD-9-CM). The Research Ethics Committee of China Medical University Hospital in Taiwan approved the study (CMUH104-REC2-115-CR-4). 

### 2.2. Study Population 

To clarify the all-cause mortality and medical burden of patients with COPD aged ≥20 years following stroke episodes, we defined four subgroups in this study, namely patients with COPD (total COPD; ICD-9 CM: 491, 492, 494, and 496), patients with COPD with stroke (ICD-9 CM: 430–438), with COPD without stroke, and those without COPD and without stroke as comparisons from 2000 to 2012. The date patients underwent a stroke event was defined as the index date in both the COPD subgroups with and without stroke. The total COPD subgroup was 1:2 frequency matched with the comparisons by sex, age, and index year. 

The primary outcomes measured in this study were hospital-based all-cause mortality, average number of hospitalization days, and frequency of medical visits. The comorbidities included histories of head injury, coronary artery disease, diabetes mellitus, hypertension, hyperlipidemia, and aspiration pneumonia. Comorbidities were defined as at least two outpatient visits or one hospitalization following the diagnosis of any comorbidity before the index date. Patients diagnosed with stroke before the index date and those aged less than 20 years were excluded from the study. All study participants were followed up from the index date to the date of death, the date they were censored, the date they withdrew from the NHIRD, or 31 December, 2013. 

### 2.3. Statistical Analyses 

We characterized the distribution of demographic factors and comorbidities across each subgroup in our study. The difference between the total COPD and comparison subgroups for each variable was tested using the chi-square test or Fisher’s exact test for categorical variables and the *t* test for continuous variables. The Kaplan–Meier method was applied to obtain the cumulative incidence curve of mortality in the study and comparison subgroups. The difference between the cumulative incidence curves was analyzed using the log-rank test. The risk of mortality in the study and comparison subgroups was evaluated using crude and multivariate Cox proportional hazard models and expressed as hazard ratios (HRs), adjusted HRs (aHRs) and 95% CIs. Stepwise multiple linear regression analysis was performed to select the most relevant factors of average hospitalization days and frequency of medical visits per year. All statistical analyses were conducted on the basis of type I error α = 0.05 using a statistical software package (SAS, version 9.4, SAS Institute Inc., Cary, NC, USA).

## 3. Results 

We included 99,192 individuals appearing in the LHID between 2000 and 2012 in this study. There were 33,064 individuals with COPD who met study inclusion criteria, of which 3207 (9.70%) had a stroke and 29,857 (90.30%) had not. There were 66,128 control subjects without a history of stroke or COPD. Among these subgroups, the ratio of female to male patients was equivalent, and the mean age was approximately 58 years, except in the COPD with stroke subgroup, which had a mean age of 64.8 years. Compared with the comparisons, patients in the COPD subgroup had a younger average age (*P* = 0.002) and lower percentages of patients with coronary artery disease (*P* = 0.001) or hypertension (*P* = 0.001). Aspiration pneumonia was not taken into analysis due to the suppressing count of less than 10. During the 14 year follow-up period, 9.70% (3207 out of 33,064; men vs. women, 11.19% vs. 8.28%) of patients with COPD in Taiwan had a stroke. However, 2.82%, 7.79%, and 18.86% of patients who had COPD with stroke were in the respective age groups of 20 to 49 years, 50 to 64 years, and 65 years or older (Table 1). 

**Figure 1:**
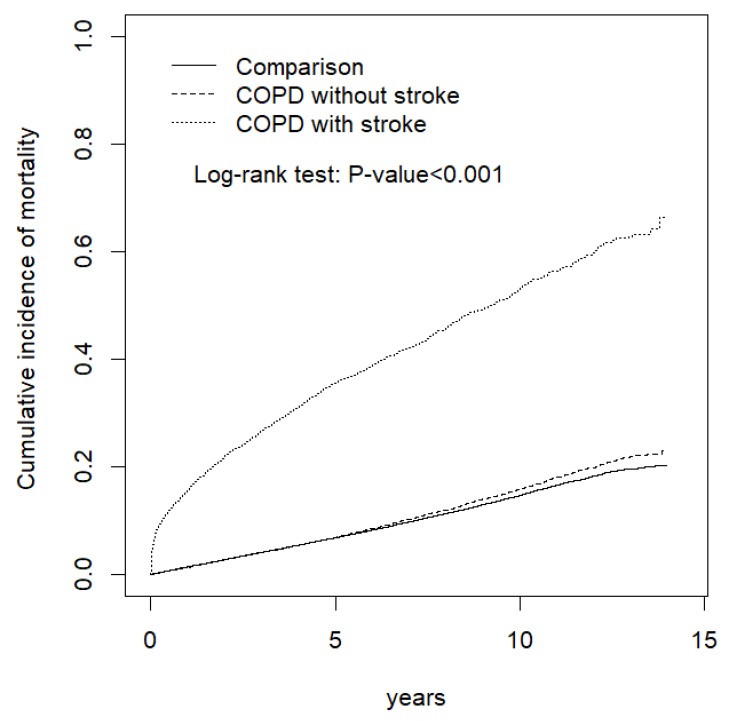
Comparison of the cumulative incidence of mortality among the COPD with stroke, COPD without stroke, and comparison subgroups.

Patients with COPD who had a stroke event had a significantly higher cumulative incidence of mortality (*p* < 0.001) compared with the patients with COPD who had no stroke event and those in the comparison subgroup (Figure 1). 

A multivariate stratification analysis was performed, and the results are presented in Table 2. Patients with COPD exhibited increased risk of mortality compared with the comparisons, including patients aged 20 to 49 years (aHR = 1.13, 95% CI = 1.15–1.53), 50 to 64 years (aHR = 1.64, 95% CI = 1.52–1.76), or more than 65 years (aHR = 1.50, 95% CI = 1.43–1.57); female patients (aHR = 1.58, 95% CI = 1.48–1.68); male patients (aHR = 1.54, 95% CI = 1.47–1.61); patients without any comorbidity (aHR = 1.52, 95% CI = 1.41–1.63); and those with one comorbidity (aHR = 1.53, 95% CI = 1.46–1.60). Patients who had COPD without stroke demonstrated increased mortality compared with the comparisons, notably patients aged 50 to 64 years (aHR = 1.28, 95% CI = 1.19–1.39) or older than 65 years (aHR = 1.17, 95% CI = 1.11–1.23), female patients (aHR = 1.22, 95% CI = 1.14–1.31), male patients (aHR = 1.25, 95% CI = 1.18–1.32), patients without any comorbidity (aHR = 1.33, 95% CI = 1.23–1.43), and those with at least one comorbidity (aHR = 1.15, 95% CI = 1.09–1.21). In the subgroup of patients who had COPD with stroke, the mortality was significantly higher than that of the comparisons, notably for patients aged 20 to 49 years (aHR = 4.73, 95% CI = 3.53–6.34), 50 to 64 years (aHR = 4.77, 95% CI = 4.26–5.33), or more than 65 years (aHR = 3.10, 95% CI = 2.90–3.32); female patients (aHR = 3.50, 95% CI = 3.19–3.84); male patients (aHR = 3.15, 95% CI = 2.93–3.39); patients without any comorbidity (aHR = 4.76, 95% CI = 4.13–5.50); and those with one or more comorbidities (aHR = 3.37, 95% CI = 3.17–3.59) (Table 2). 

Table 3 presents the incidences and HRs of mortality stratified by age, sex, and comorbidity for patients who had COPD with stroke compared with patients who had COPD without stroke. Patients who had COPD with stroke exhibited significantly higher mortality than those without stroke, especially those aged 20 to 49 years (aHR = 4.97, 95% CI = 3.57–6.91), 50 to 64 years (aHR = 3.97, 95% CI = 3.50–4.51), or more than 65 years (aHR = 2.79, 95% CI = 2.58–3.02); female patients (aHR = 3.16, 95% CI = 2.83–3.52); male patients (aHR = 2.66, 95% CI = 2.45–2.89); and patients with (aHR = 3.05, 95% CI = 2.84–3.27) and without comorbidities (aHR = 3.63, 95% CI = 3.11–4.24) (Table 3). 

The main risk factors associated with the average number of hospitalization days per year are presented in Table 4. Patients who had COPD without stroke, patients who had COPD with stroke, older patients, male patients, and patients with diabetes, head injury, or coronary artery disease had a higher average number of hospitalization days per year. However, patients with hyperlipidemia had a lower average number of hospitalization days per year in this study (Table 4). 

Table 5 presents the main factors associated with the frequency of medical visits per year. Patients who had COPD without stroke, patients who had COPD with stroke, older patients, and patients with diabetes, hypertension, hyperlipidemia, head injury, or coronary artery disease had a higher frequency of medical visits per year. By contrast, male patients had a lower frequency of medical visits per year in this study (Table 5). 

## 4. Discussion

COPD is currently recognized as a complex multicomponent disease and frequently coexists with other disorders because of chronic systemic inflammation at any stage of the disease progression [13]. Therefore, it could increase either the risk of developing stroke or the all-cause mortality risk in patients. Our results demonstrated that Taiwanese adults with COPD exhibited only a slight increase in all-cause mortality compared with the comparisons during the 14 year follow-up. After a stroke episode, the all-cause mortality risk in patients with COPD exhibited a substantial 3.11- to 4.82-fold increase compared with that of the comparisons and 2.66- to 5.05-fold increase compared with that of patients with COPD without stroke. When comparing our study results with those of a similar study also conducted using the NHIRD [14], we noticed that patients with COPD aged ≥ 65 years consistently had a higher prevalence of stroke, but the poststroke mortality risk was higher in the younger ones. 

Although some authors have proposed that COPD could be the third leading cause of death globally [15], this study provides additional evidence that COPD with stroke is much more lethal than COPD alone. Numerous comorbid or coexisting disorders have been reported to accompany COPD, including cardiovascular diseases, metabolic disorders, bone and skeletal muscle dysfunction, mental and cognitive impairment, gastrointestinal diseases, and other respiratory disorders such as asthma, bronchiectasis, pulmonary fibrosis, and lung cancer [13,16,17,18]. COPD usually interacts with other comorbidities, such as stroke, aspiration pneumonia, diabetes mellitus, head injury, or hypertension, thus predisposing patients to poorer long-term survival. Remoortel et al. analyzed and reported considerable interactions of cardiovascular diseases with smoking habits and physical inactivity in patients recently diagnosed with COPD and in smokers without COPD [18]. Their results indicated that COPD should be considered one of multiple risk factors rather than a single mortality cause for patients. As we currently know, patients with COPD, even those with a clinically stable condition, have higher circulating levels of C-reactive protein, fibrinogen, interleukin-6, leukocytes, and tumor necrosis factor alpha [19,20]. This indicates that oxidative stress and low-grade systemic inflammation play notable roles in the common pathogenesis of COPD and stroke besides smoking habit and physical inactivity. However, the mechanistic interactions between COPD and stroke require more detailed investigation to understand it fully. 

In this study, we enrolled patients who had both first-time and recurrent strokes to estimate all the possible correlations between COPD and poststroke outcomes in patients with COPD. When patients survive an acute stroke episode, various disabilities usually result, increasing the medical burden on families and health care systems [21,22]. This study revealed that COPD with stroke, diabetes mellitus, head injury, COPD without stroke, and male sex are leading factors contributing to the increase in average number of hospitalization days. The enhancing effect of hospitalization was particularly high for COPD with stroke. For the increase in frequency of medical visits, the leading influential factors were COPD with stroke, COPD without stroke, diabetes mellitus, hypertension, and coronary artery disease. Female patients visit the hospital more frequently than male patients do in Taiwan. The sex differences observed among the Taiwanese population when it comes to seeking medical advice are similar to those from previous reports [23,24]. However, our results reaffirm that pulmonary disorders should be aggressively treated and controlled to reduce the medical burden in this century. Because cardiovascular disorders and cancers have been monopolizing considerable resources from our health care system, the government should consider the rising prevalence of COPD and its correlations with other disorders [3]. 

Because this study included a nationwide, population-based sample with little risk of recall or selection bias, our findings provide valid information regarding the effect of stroke on the mortality risk of patients with COPD. However, further studies in different countries are warranted to address this matter in different countries and societies, given the possible confounding effects of ethnic, cultural, socioeconomic, and administrative differences around the world. First of all, we could not directly contact our patients or their families to obtain individual details because their identities were anonymized in the NHIRD. The study design did not take into account the financial burden on the patients’ families or the type and severity level of the patients’ COPD and stroke. As we known, it is hard to say whether COPD in severer status is associated a higher risk for developing stroke by current evidences [11,25]. However, those could be confounding factors for a higher mortality risk in patients with COPD with or without stroke. We would like to recognize this study limitation as the major one. Second, our dataset only included the inpatient all-cause mortality of patients with COPD with or without stroke based on the medical coding system used in Taiwan. The individual mortality rates and causes of deaths that occurred outside the hospital were not included in this study. The NHI program is a mandatory insurance program and covers more than 99% of population in Taiwan. All of the residents are guaranteed to have equal access to medical service. This health care system has few disparities in access to inpatient services across different areas in Taiwan regardless of their background, socioeconomic status, and critical illness status [26,27]. Therefore, the proportion of patients with COPD or stroke dying outside hospitals is low in this study. Third, although the NHI administration performs thorough quarterly reviews and false claims are heavily sanctioned to ensure the accuracy of the NHIRD, miscoding can always occur, although it is unlikely. However, our results did indicate that the sample size was sufficient to statistically estimate the mortality risk and medical burden in patients who had COPD with or without subsequent stroke. 

## 5. Conclusions 

COPD is a multidimensional heterogeneous disorder encompassing both pulmonary and extrapulmonary comorbidities. The mortality risk in patients with COPD is evidently increased by stroke. However, the poststroke mortality risk is reciprocally increased if the patients are younger. Moreover, the average number of hospitalization days and frequency of medical visits are substantially increased in patients who have COPD with stroke. Our findings indicate that additional efforts are warranted in the protection of patients with COPD from multiple ill conditions to reduce their overall mortality and medical burden. Further studies are warranted to address this matter in different countries around the world. 

## Figures and Tables

**Table 1:** Distributions of age, sex, and comorbidity in the chronic obstructive pulmonary disease (COPD) and comparison subgroups.

	COPD						Comparison		
	Total N = 33,064		COPD without Stroke N = 29,857		COPD with Stroke N = 3207		N = 66,128		
	n	%	n	%	n	%	n	%	*p*-value †
Age, year									0.001
20–49	7986	24.2	7761	26.0	225	7.02	14,917	22.6	
50–64	15,795	47.8	14,564	48.8	1231	38.4	31,753	48.0	
≥65	9283	28.1	7532	25.2	1751	54.6	19,458	29.4	
Mean (SD) ^§^	58.1	10.0	57.4	9.82	64.8	9.42	58.3	10.3	0.002
Sex									0.40
Women	16,551	50.1	15,180	50.8	1371	42.8	32,915	49.8	
Men	16,513	49.9	14,677	49.2	1836	57.3	33,213	50.2	
Comorbidity									
Head injury	1138	3.44	953	3.19	185	5.77	2187	3.31	0.27
Coronary artery disease	6510	19.7	5536	18.5	974	30.4	13,844	20.9	0.001
Diabetes mellitus	2864	8.66	2119	7.10	745	23.2	5684	8.60	0.72
Hypertension	14,865	45.0	12,383	41.5	2482	77.4	30,657	46.4	0.001
Hyperlipidemia	10,874	32.9	9651	32.3	1223	38.1	21,690	32.8	0.78

Chi-square test; ^§^ *t* test; † Total COPD vs. comparison.

**Table 2:** Incidence and hazard ratio of mortality stratified by age, sex, and comorbidity in the COPD and comparison subgroups.

Variables	Comparison N = 66,128			COPD								
				Total N = 33,064			COPD without Stroke N = 29,857			COPD with Stroke N = 3207		
	Event no	Rate	aHR ^‡^ (95% CI)	Event no	Rate	aHR ^‡^ (95% CI)	Event no	Rate	aHR ^‡^ (95% CI)	Event no	Rate	aHR ^‡^ (95% CI)
Age group (years)												
20–49	500	4.82	1(Reference)	329	6.13	1.33(1.15, 1.53) *	274	5.24	1.15(1.00,1.34)	55	38.2	4.73(3.53, 6.34) *
50–64	1787	8.79	1(Reference)	1372	14.0	1.64(1.52, 1.76) *	972	10.7	1.28(1.19, 1.39) *	400	56.5	4.77(4.26, 5.33) *
≥65	4567	32.8	1(Reference)	2990	49.2	1.50(1.43, 1.57) *	1929	37.7	1.17(1.11, 1.23) *	1061	109.4	3.10(2.90, 3.32) *
Sex												
Women	2637	12.0	1(Reference)	1687	16.0	1.58(1.48, 1.68) *	1114	11.4	1.22(1.14, 1.31) *	573	71.3	3.50(3.19, 3.84) *
Men	4217	18.6	1(Reference)	3004	28.1	1.54(1.47, 1.61) *	2061	21.3	1.25(1.18, 1.32) *	943	92.7	3.15(2.93, 3.39) *
Comorbidity												
None	1936	12.2	1(Reference)	1249	16.0	1.52(1.41, 1.63) *	1039	13.6	1.33(1.23, 1.43) *	210	116.5	4.76(4.13, 5.50) *
With any one	4918	17.1	1(Reference)	3442	25.6	1.53(1.46, 1.60) *	2136	18.1	1.15(1.09, 1.21) *	1306	79.6	3.37(3.17, 3.59) *

Rate, per 1000 person-years; aHR, adjusted hazard ratio; ^‡^ multivariable analysis including age, sex, and comorbidities (head injury, coronary artery disease, diabetes mellitus, hypertension, and hyperlipidemia). * *P* < 0.001.

**Table 3:** Incidence and hazard ratio of mortality stratified by age, sex, and comorbidity in patients who had COPD with stroke compared with those who did not have stroke.

	COPD without Stroke N = 29,857 aHR ^†^ (95% CI)	COPD with Stroke N = 3207 aHR ^†^ (95% CI)
All	1.00	2.83(2.64, 3.02) *
Age, year		
20–49	1.00	4.97(3.57, 6.91) *
50–64	1.00	3.97(3.50, 4.51) *
≥65	1.00	2.79(2.58, 3.02) *
Sex		
Women	1.00	3.16(2.83, 3.52) *
Men	1.00	2.66(2.45, 2.89) *
Comorbidity		
None	1.00	3.63(3.11, 4.24) *
With any one	1.00	3.05(2.84, 3.27) *

Rate, per 1000 person-years; aHR, adjusted hazard ratio; ^‡^ multivariable analysis including age, sex, and comorbidities (head injury, coronary artery disease, diabetes mellitus, hypertension, and hyperlipidemia). * *P* < 0.001.

**Table 4:** Stepwise regression analysis of the average number of hospitalization days per year (all-cause admission).

Variable	Parameter Estimate	Standard Error	95% CI
COPD without stroke vs. Comparison	1.39	0.16	(1.08, 1.70) *
COPD with stroke vs. Comparison	49.2	0.42	(48.4, 50.0) *
Age (every one year)	0.18	0.01	(0.16, 0.19) *
Sex (male vs. female)	1.29	0.15	(1.00, 1.58) *
Diabetes mellitus	4.85	0.26	(4.34, 5.37) *
Hyperlipidemia	−2.33	0.16	(−2.64, −2.01) *
Head injury	2.23	0.40	(1.43, 3.02) *
Coronary artery disease	0.76	0.18	(0.40, 1.12) *

* *P* < 0.001.

**Table 5:** Stepwise regression analysis of the frequency of medical visits per year.

Variable	Parameter Estimate	Standard Error	95% CI
COPD without stroke vs. Comparison	8.29	0.15	(8.00, 8.58) *
COPD with stroke vs. Comparison	24.4	0.38	(23.6, 25.1) *
Age (every one year)	0.22	0.01	(0.21, 0.24) *
Sex (male vs. female)	−3.39	0.13	(−3.65, −3.13) *
Diabetes mellitus	8.16	0.24	(7.68, 8.64) *
Hypertension	6.40	0.15	(6.11, 6.70) *
Hyperlipidemia	5.43	0.15	(5.14, 5.72) *
Head injury	4.24	0.37	(3.52, 4.97) *
Coronary artery disease	6.06	0.17	(5.73, 6.40) *

* *P* < 0.001.

## Abbreviations

NHIRD	National Health Insurance Research Database;
aHR	adjusted hazard ratio;
ICD-9-CM	International Classification of Diseases, Ninth Revision, Clinical Modification
